# Compliance of Physiotherapeutic Scoliosis-Specific Exercise in Adolescent Idiopathic Scoliosis: A Scoping Review

**DOI:** 10.3390/jcm14092950

**Published:** 2025-04-24

**Authors:** Azharuddin Fazalbhoy, Jeb McAviney, Rosemary Mirenzi

**Affiliations:** 1 School of Health and Biomedical Sciences, RMIT University, Melbourne, VIC 3000, Australia; azharuddin.fazalbhoy@rmit.edu.au; 2 ScoliCare, Suite 5.08, Level 5/15 Kensington St, Kogarah, Sydney, NSW 2217, Australia

**Keywords:** adolescent idiopathic scoliosis, physiotherapeutic scoliosis-specific exercises, compliance, adherence, non-surgical management

## Abstract

**Background**: Non-surgical management of adolescent idiopathic scoliosis (AIS) includes physiotherapeutic scoliosis-specific exercise (PSSE). Determining the efficacy of PSSE in AIS has been challenging as the clinical effect is closely linked to exercise compliance (a dose–response relationship), with home exercise programs (HEPs) showing a general trend for decreased participation over time. The existing literature reports contradictory definitions and parameters of appropriate PSSE compliance in AIS. As such, this scoping review is necessary for therapists to identify PSSE prescription consistency, enabling clear guidelines for improved PSSE compliance. **Methods**: A scoping review of the literature was conducted to synthesize original research from inception to November 2024 and reference lists were examined for studies reporting compliance, adherence, or motivational strategies for PSSE in AIS. **Results**: Fifteen articles with a focus on PSSE in AIS were included in this review. The results demonstrate that compliance (C) and adherence (A) are terms commonly used interchangeably, only half of the studies clearly define compliance and/or adherence, and some utilize motivational strategies; however, outcomes of compliance/adherence were often not reported in the Results section or reflected in the discussion of results. **Conclusions**: Compliance and/or adherence are inconsistently reported within studies and numerous variations exist in (1) the section of the paper it is mentioned, (2) whether the inclusion criteria hinged on patient compliance/adherence, and (3) whether motivational strategies were employed and outcomes reported. Notably, there was a lack of compliance or adherence reporting in Results and Discussion sections of papers. The definition of appropriate compliance and any effective motivational strategies to improve compliance to achieve the desired results for treatment of AIS remain undetermined.

## 1. Introduction

Adolescent idiopathic scoliosis (AIS) is a three-dimensional deformity of the spine that affects 1–3% of children aged 10–16 years old [1]. The condition is characterized by a lateral curvature of the spine greater than 10°, measured by Cobb angle, on a posterio-anterior radiograph [2], combined with vertebral rotation [3]. The risk of curve progression is more common in females, and they are up to seven times more likely to have a curve greater than 40° [4]. While a very small percentage of individuals with AIS are at risk of requiring surgical intervention, bracing is widely accepted as the primary non-surgical intervention method for curves between 25 and 40° [5]. Physiotherapeutic scoliosis-specific exercises (PSSEs) are also prescribed as an adjunct to bracing for curves between 25 and 40° or a stand-alone intervention for mild curves measuring less than 20° [5].

To date, several schools and methods of PSSE have emerged across the globe which have distinctive elements structured within their scoliosis-specific exercise programs. In 2016, The International Society on Scoliosis Orthopedic and Rehabilitation Treatment (SOSORT) established common principles and guidelines incorporated by every PSSE school and method, ranking “active self-correction in 3D” as an important element to be included in the exercise programs by consensus [5]. Active self-correction in 3D can be defined as the search for the best possible alignment the patient can achieve in the three spatial planes. Numerous methodologically sound randomized clinical trials have demonstrated improved clinical and radiographic measures following PSSE training [6,7,8]. More recently, a retrospective study demonstrated that patients undergoing PSSE and bracing could also improve their trunk muscle endurance [9].

A significant factor in non-surgical intervention is patient compliance with recommended regimens, and a lack of compliance can result in suboptimal clinical effect. A recent study reporting long-term compliance with a PSSE program using a well-defined AIS population and clear comparison groups (those completing ≥10 PSSE sessions versus those completing fewer) comprehensively evaluated PSSE compliance and reported a general trend for decreased participation in prescribed PSSE home exercise programs (HEPs) [1]. While the limitations of this study were its observational design and potential bias in self-reported data, it indicates challenges in determining efficacy and a dose–response relationship of PSSE in AIS, creating uncertainty for therapists prescribing HEPs in the real-world if parameters that define PSSE compliance in AIS, motivational strategies to increase compliance, and the real-world impact on treatment prescription and modification are unknown. This is in contrast to robust bracing intervention studies using both randomized and preference cohort study designs, where it is well established that an increased number of hours of brace wear is associated with better clinical outcomes [10]. Furthermore, when compliance is mentioned in published PSSE studies, seldomly are they factored into any treatment modifications or results of the study. The objective of this scoping review is to gain an understanding of consistency in the reporting of compliance or adherence within studies of PSSE in AIS and identify motivational strategies employed by investigators to successfully enhance compliance to these programs.

## 2. Materials and Methods

### 2.1. Methodological Basis

A scoping review was performed to examine the consistency in reporting of compliance or adherence of PSSE in AIS and identify motivational strategies, if any were employed to successfully enhance compliance to these programs. The review was conducted using methods specified in the Preferred Reporting Items for Systematic Reviews and Meta-Analyses extension for Scoping Reviews (PRISMA-ScR) statement for reporting. The methodological framework was based on previously published work by Arskey and O’Malley [11].

### 2.2. Data Sources

The overall search strategy was designed to capture a wide breadth of literature related to the research questions guiding this scoping review: How consistently are compliance and adherence of PSSE reported in the published literature? Are motivational strategies used to enhance compliance and adherence consistently reported? If so, are they factored into the result of the study? Selected medical subject heading (MeSH) terms and keywords from PubMed were combined with Boolean operators (e.g., Or, AND) to develop the search strategy. Proceeding with this strategy, a structured literature search was conducted using three scientific databases: PubMed, Cumulative Index to Nursing and Allied Health Literature (CINAHL), and Excerpta Medica database (EMBASE) to identify relevant studies from inception to October 2023 and updated in November 2024.

### 2.3. Search Terms

The search terms used include the following: (1) treatment adherence and compliance (MeSH), patient adherence, client adherence, patient non adherence, patient compliance (MeSH), therapeutic compliance, client compliance, patient cooperation, motivators; (2) physiotherapeutic scoliosis specific exercise, PSSE, scoliosis specific exercise, scientific exercise approach to scoliosis, SEAS, Schroth; (3) scoliosis (MeSH), adolescent idiopathic scoliosis, spinal curvatures (MeSH), spinal deformity, Scheueramanns (MeSH). The detailed search strategy used for the database PubMed has been provided as an example (Appendix A).

### 2.4. Eligibility Criteria

All studies retrieved through the search strategy were screened using the following inclusion criteria: (1) reported on adolescent idiopathic scoliosis, (2) included therapist-prescribed exercise-based rehabilitation, (3) published in English, and (4) reported on enablers, motivators, challenges, and barriers. Studies were excluded if they did not report on adolescent idiopathic scoliosis, reported on bracing or combined treatments of bracing and exercise, study participants had a history of spinal surgery, or if they did not report compliance or adherence. Letters to the editor, opinion pieces, and reviews, including systematic reviews, were excluded; however, a screening of reference lists of systematic reviews was conducted.

### 2.5. Identifying Relevant Studies

A two-stage screening process was undertaken by the authors. All search results were initially imported to an EndNote 21 library accessible to all reviewers. Author AF reviewed all titles identified by the search results and discarded the irrelevant studies. Both reviewers (AF, RM) then independently reviewed relevant titles and abstracts. Full-text articles were then further reviewed independently by both reviewers and any conflicts were resolved through discussion. If discussions failed to achieve a consensus, JM was available for resolution.

### 2.6. Quality Appraisal

Aligning to the guidelines of a scoping review, no formal assessment of the methodological quality of the included studies was performed.

### 2.7. Data Extraction and Analysis

Full-text articles were analyzed to draw out relevant information on participant compliance and adherence to the therapist-prescribed exercises. Each section of the full-text articles was carefully analyzed to search for reporting on the compliance or adherence to the therapist-prescribed exercise or home exercise program. Both AF and RM screened through full-text articles and categorized them into compliance or adherence. The articles were reviewed for usage of the word compliance or adherence.

## 3. Results

### 3.1. Search Results

**Figure 1:**
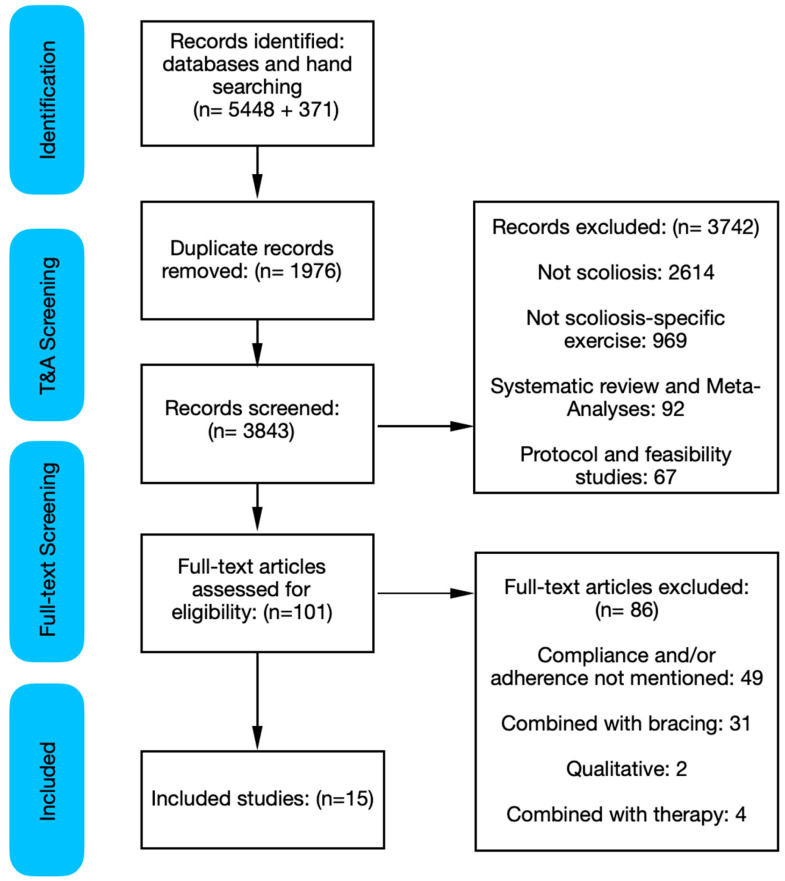
Preferred Reporting Items for Systematic Reviews and Meta-Analyses flow diagram. T&A: title and abstract. Initial search from inception to October 2023 identified 5448 records. An updated search in November 2024 identified a further 371 records.

A total of 5448 articles were retrieved for this scoping review using the search strategy from databases and hand searching. A further 371 articles were retrieved when the search was updated (29 November 2024). Duplicate records were removed (*n* = 1851 + 125) and the remaining records (*n* = 3597 + 246) were screened for the purpose of the review. After screening, 3517 + 225 records were removed (2457 + 157 not scoliosis, 924 + 45 not scoliosis-specific exercise, 74 + 18 systematic reviews or meta-analyses, 62 + 5 protocol or feasibility study) and the remaining 80 + 21 records underwent full-text screening. After full-text screening, a total of 15 articles were included in the analysis for data extraction. (Figure 1). A complete list of all included studies is outlined in Table 1.

### 3.2. Compliance and Adherence Reporting

Out of the 15 articles, the use of terms such as compliance (C) was used eight times [1,7,12,17,18,20,22,23] and adherence was used five times [13,15,19,21,24] in the text of the article (C: ~53%; A: ~33%). It was used interchangeably in two articles (both: ~13%) [14,16]. This indicates a preference by authors to report exercises being performed as intended following the prescribed regimen accurately over maintaining commitment and adherence. Further elaboration on parameters as to what comprises compliance or adherence was mostly reported in the articles (parameters defined: 67%; not defined: 33%). This permits objective assessment of the intervention reported, control for variations in participation, and establishing meaningful benchmarks necessary for outcomes. The section of the article where these terms appeared was variable and inconsistent (Introduction, one article [1]; Methodology, twelve articles [7,12,13,14,15,16,17,20,21,22,23,24]; Results, one article [17]; and Discussion, one article [18]). This suggests that most investigators recognize the importance of incorporating PSSE compliance and/or adherence within study designs; however, seldomly are they reporting how compliant participants are in the Results nor do they contextualize it further in the Discussion.

### 3.3. Motivational Strategy Reporting

From the fifteen articles included in the current study, five articles used motivational strategies to engage participants in their program (strategy: 33%; no strategy: 67%) [13,14,18,22,24]. The strategies varied from using questionnaires completed by participants, guardians, and therapists [13,14], to closed chat groups [18], and weekly email check-ins [24]. Four articles [13,14,22,24] described their motivational strategy and reported them in the Results section and Discussion (reported and discussed: 33%; not reported or discussed: 67%). Motivational strategies can foster intrinsic motivation, increase self-efficacy, and reduce dropout rates, which can significantly enhance intervention effectiveness and improve data collection and reporting.

## 4. Discussion

This scoping review demonstrates variability and inconsistency in the reporting of PSSE compliance and adherence in AIS studies analyzing the efficacy of PSSE. Of the studies that do report compliance or adherence, there appears to be no clear definition of these terms, what constitutes ‘compliance’ or ‘adherence’ in a PSSE program, and in some instances, the terms are used interchangeably by authors. The lack of attention to reporting PSSE compliance or adherence presents significant challenges for clinicians and researchers in determining the efficacy of PSSE programs, providing evidence-based guidance for PSSE programs, and understanding the dose–response relationship to modify treatment prescription.

From a definition standpoint, the words compliance and adherence have differing meanings. Exercise compliance can be defined as performing a set of exercises in their mode, type, duration, and frequency as prescribed by the treating therapist. The patient completes the prescribed exercises as instructed by the treating therapist—“doing as they are told”. Whereas adherence can be defined as maintenance of an active involvement in prescribed exercise and the extent to which the behavior of a person correlates with the agreed plan of the suggested exercise intervention [25]. While definitions appear to overlap, the latter emphasizes a proactive choice of patients to follow through with the prescribed treatment and relates to the degree to which the target intensity and volume are achieved [26]. Using the terms interchangeably, as demonstrated by this review, without clearly defining these terms leads to inaccurate reporting and descriptions of how patients managed to follow the prescribed intervention. Based on these definitions, it would be reasonable to suggest future studies report on one or both terms with clear definitions so clinicians can determine the reality in everyday clinical practice.

Inconsistencies in what constitutes compliance or adherence were also evident in this review. For example, Dufvenberg et al. [13] utilized a self-reported questionnaire consisting of three questions related to patient adherence, motivation, and capability. The treating therapist and patient were also asked to report, using a 4-point scale—from best “very sure” (1 point) to worst “not at all” (4 points)—how they feel they have completed the treatment plan. These data were collected only once at a six-month follow-up consultation and risks confounding factors influencing treatment adherence and variability appeared in the outcome reporting. However, Mohamed et al. [19] considered adherence as attendance of treatment sessions prescribed by the treating therapist—one hour, three times a week, over the same timeframe of six months. Using these parameters, they reported patient adherence as 98%, although the study’s small sample size limit generalizability. In contrast, an observational study by Negrini et al. [20] reported exercise compliance rate by dividing the number of exercise sessions patients performed by the expected frequency, which was two sessions per week. Using this approach, they observed patients completed 48 min of exercise per session with 95% compliance; however, the generalizability of findings could be limited by the small sample size. The variability in these parameters relating to exercise compliance and adherence raises questions about what the minimum dosage of prescription of a PSSE program that would benefit the patient with an acceptable level of burden to the child, the family, and the local medical system would be.

Few studies utilized or reported any motivational strategies for ensuring compliance and/or adherence of PSSE programs. Methods to motivate patients varied significantly, ranging from emailing questionnaires, checklists to be completed by the patient about their exercises every week or two weeks [14,24], to using mobile phone applications such as WeChat [18]. While most studies in this review failed to utilize any motivational strategies, when they were utilized, the outcomes of such strategies were not reported in the Results or Discussion; therefore, the effectiveness of such strategies was not clear. This makes it challenging to draw any conclusions about the utilization of motivational strategies to enhance compliance and/or adherence. Without adequate motivation, participants may fail to follow through with the required frequency, intensity, or duration of the exercise intervention. This is particularly critical in analyzing dose–response relationships between the intervention and outcomes. It is well established that participation and adherence in rehabilitation programs for conditions such as strokes can be enhanced by utilizing motivational strategies and concepts [27].

Studies that set clear parameters for PSSE compliance and/or adherence often failed to report these in the Results section of the paper and/or discuss them as part of their overall findings. The study by Gao et al. [15] outlined a home exercise program for patients that included 1 h of exercise, two to three times a week. Patients also completed a 14-day intensive training during vacation time program supervised by certificated physical therapists. They concluded that for AIS patients with a Risser 3–5 and a Cobb angle 20–40°, Schroth exercises improved HRQOL and halted curve progression. However, the extent to which patients complied with the treatment plan was not reported in the Results. The lack of reporting presents significant challenges to determine whether the dose response for patients was at an acceptable level.

The other prominent issue relevant to this review is that most research papers only focus on the application of one method of PSSE, with a one-size-fits-all exercise program, without factoring in the reality of everyday clinical practice. Marchese et al. [28] reported that PSSE therapists are often likely to mix their methods and accommodate the program to suit the individual patients and their needs. The more methods the therapist was trained in, the more likely the therapist was to use a variety of methods for each patient. Recently, Marchese et al. [9] published a study that described the ScoliBalance approach to PSSE, which encompasses a mix of strategies from a variety of PSSE methods to demonstrate the benefits on an individualized approach. Future studies should focus on the reality of everyday practice and the reality of motivating a young patient to do these exercises for extended periods of time to achieve acceptable results.

To date, reliable data that clearly demonstrate the minimum compliance required for a positive clinical outcome remain lacking. Establishing a clear dose–response relationship for effective clinical outcomes is fundamental for guiding treatment strategies. Achieving a consensus on the precise definitions and parameters of PSSE compliance is the essential first step in this process, as it will lay the groundwork for determining the minimum dose required to achieve measurable clinical benefits and ensure the effective application of exercise interventions. Without this clarity, the relationship between exercise dosage and clinical improvement remains poorly understood, limiting the ability to tailor interventions and predict outcomes with confidence.

There are some limitations in this scoping review that must be addressed. In our attempt to focus on studies that included scoliosis-specific exercise programs in the management of adolescent idiopathic scoliosis, some studies may have been overlooked or excluded due to our search parameters. While a rigorous search strategy was developed and refined to yield a breadth of studies and we used relevant journal databases and hand-searched references for inclusion, we did not register or publicly post the review protocol prior to searching, nor did we contact experts in the field to enquire about key studies that should be included in our review. Additionally, as per scoping review methodology, no quality appraisal was conducted on included studies to ensure a broad overview of evidence was achieved. The aims and objectives of the review remained central to our search which may have excluded some studies.

## 5. Conclusions

Compliance and/or adherence of PSSE programs are mentioned inconsistently and with variability within studies investigating the efficacy of PSSE in AIS. This review highlights numerous variations in (1) the section of the paper in which compliance or adherence is mentioned, (2) whether or not the inclusion criteria hinged on patient compliance, (3) whether motivational strategies were used and reported, (4) whether the impact of motivational strategies on outcomes was reported, and if so, whether treatment was adapted in any way to improve compliance/adherence. Most notable in this review was the lack of compliance or adherence reporting in the Results and Discussion sections of the paper, leading to some ambiguity about the dose–response relationship of PSSE in the reality of everyday practice. The definition of appropriate compliance, and any effective motivational strategies to improve compliance, of PSSE in AIS to achieve maximum, or even minimum, desired results for treatment of AIS remains undetermined.

## Figures and Tables

**Table 1:** Summary of studies reporting compliance and/or adherence, section of the paper they are reported in, and motivational strategies employed.

Study Identifiers: First Author (year), Category (Sample Size) [Reference]	Parameters of Compliance or Adherence Defined and Reported	Section of the Paper Mentioning	Motivational Strategies	Factored into Results	Factored into Discussion
Büyükturan et al. (2024), Compliance (*n* = 31) [12]	Supervised exercise sessions of 90 min each, three times a week for six months. If patient compliance was below 70%, precautions were taken. Home exercises consisted of stretching, posture training, breathing, and spinal flexibility exercises. Frequency and duration unclear.	Mentioned in the Materials and Methods section of the paper.	Not reported	Authors mentioned the study was completed with 100% attendance. No further information provided.	Not reported
Dufvenberg et al. (2021), Adherence (*n* = 135) [13]	A 4-point scale—from best “very sure” (1 point) to worst “not at all” (4 points)—was used to report adherence, motivation, and capability. The patient was asked to self-report adherence “the grade to which you feel that you have completed the treatment”.	The importance of adherence mentioned in the Methods.	Employed motivational strategies using the capability, opportunity, motivation, and behavior change model.	Not reported	Authors reported the level of participant adherence and compliance to treatment reflecting upon the outcomes of the treatment.
Fan et al. (2024), Compliance and Adherence (*n* = 763) [14]	Reported compliance. Described as 1 h private session a week, 1 h group session on weekends, and 45–60 min at home daily.	Reported in Materials and Methods under sub-section PSSE Protocol and Compliance.	Participants were auto-sent a checklist every two weeks gathering data about compliance, hours per day. Self-reported data.	Yes, reported in the results. Analysis shows the daily compliance and non-compliance. Scoliosis curve regression related to exercise compliance.	Yes, reported in the Discussion. Authors highlighted the impact on compliance and non-compliance on the outcomes reported in the Discussion.
Gao et al. (2021), Adherence (*n* = 64) [15]	Home exercise program adherence was set to two or three times per week for 1 h. Participants completed a 14-day intensive training during vacation time program supervised by certificated physical therapists.	Adherence mentioned in the Methods section under sub-headings Inclusion criteria “Inclusion criteria and exclusion criteria of Schroth group” and “Schroth exercise treatment”.	Not reported	Not reported	Not reported
Karavidas et al. (2024), Compliance and Adherence (*n* = 163) [16]	Every patient was advised to perform exercises at home 5 times per week, for 30 min. supervised sessions took place in our clinic once a week, for 55 min. Compliance was self-reported monthly using a scale from A to C (A = 5 d p/w, B = 3–4 d p/w, C = less than 2 d p/w).	Treatment protocol section under Materials and Methods	Not reported	Reported in the Results section. Compliance was good, having 78 subjects (47.9%) with excellent (A), 54 subjects (33.1%) with moderate (B), and 31 subjects (19%) with poor compliance (C).	Compliant patients had significantly less progression rate and brace prescription. Authors suggest it is attributed to the clearly described treatment protocol and to the regular supervised sessions and clinical follow-up, providing motivation.
Kocaman et al. (2021), Compliance (*n* = 28) [17]	Compliance is not clearly outlined. A vague statement about the study being completed with 100% compliance is mentioned.	Results—a brief mention of the study being completed with 100% compliance.	Not reported	Not reported	Not reported
Kuru et al. (2016), Compliance (*n* = 45) [7]	Reported compliance. The parameters of what constitutes compliance was not defined; however, authors checked with caregivers whether the exercises were regularly performed.	Mentioned in the Methods section of the paper.	Not reported	Not reported	Not reported
Liu et al. (2020), Compliance (*n* = 99) [18]	No parameters set for compliance.	A brief mention of compliance mentioned in the Discussion. Authors mention using the WeChat app to ask the number of hours per day participants exercised.	Authors highlight the use of a chat app to keep participants motivated.	Not reported	Not reported
Mohamed et al. (2021), Adherence (*n* = 34) [19]	Adherence is not clearly outlined. Attendance of treatment sessions was 98%, as mentioned in the Methods section of the intervention.	Methods—interventions	Not reported	Not reported	Not reported
Negrini et al. (2008), Compliance (*n* = 74) [20]	Compliance rate by dividing the number of exercise sessions performed by the expected frequency of two sessions per week. The patients continue treatment at a rehabilitation facility near their home (by themselves or with their parents) twice a week (40 min per session) plus one daily exercise at home (5 min).	Compliance is mentioned in the Material and Methods section. It is more specifically mentioned under the sub-heading “treatment” and under the sub-heading “outcome measures”.	Not reported	Reported in the Results section. Authors describe the percentage of compliance as 95%.	Discussion mentions analysis undertaken to determine compliance of greater than or equal to 30 min of exercise and 10 min or less as exercise curve progression.
Negrini et al. (2019), Adherence (*n* = 327) [21]	As part of home exercises for SEAS, an agenda set for adherence. Defined as 90 mins per week according to patient preferences. Therapist sessions set at four per year, once every three months. For usual physiotherapy group, adherence is assessed through self-reporting of participants and families.	Described in the Methods section of the paper. Protocol of intervention states adherence and parameters of adherence.	It is not apparent that strategies for improved adherence were employed.	Adherence of exercise is not reported. Rates of failure and dropout have been reported; however, it is unclear about rate of exercise adherence.	Participant adherence to exercise outlined in Discussion. Authors detail the number of minutes and standard deviation of participant adherence.
Simhon et al. (2021), Compliance (*n* = 81) [1]	HEP compliance, which was defned as performing ≥ 80 min of home exercises per week. Caregivers asked to recall number of minutes and days practised per week of home exercise program at four time points of interest: 1 week, 3 months, 1 year, and 2 years.	Mentioned in the Introduction as a factor for success of non-operative intervention. Also highlights the limitations of the published literature on tracking compliance of home exercise programs.	Not apparent if motivational strategies were used to motivate participant compliance.	Reported as the percentage of patients that were included in the study who were complaint with the defined parameters of compliance at each time point.	Discussed as the primary outcomes of the study. Details the mean number of minutes of home exercise compliance at the four different time points.
Tombak et al. (2024), Compliance (*n* = 37) [22]	Supervised exercise for one hour twice a week for twelve weeks. Participants in this group exercised at home for the remaining one hour five days a week. Another group home program consisted of unsupervised exercises for about 1 h every day for 12 weeks. Parameters were set to 83%.	Interventions under Materials and Methods section of the paper.	Home equipment and access to facilities were provided; it was made fun by technology support/video recording, and parent involvement was encouraged.	Exercise compliance performed with a physiotherapist was 100%, and home exercise compliance was 97.54%. In the home group (HSEG), compliance with the home program was 96.89%.	Not reported
Zapata et al. (2019), Compliance (*n* = 49) [23]	Compliance is not clearly outlined. The exclusion criteria list developmental disorders that prevent understanding and compliance with the exercise schedule.	Methods—listed as an exclusion criteria. Participants excluded if they have developmental disorder preventing exercise compliance.	Not reported	Not reported	Not reported
Zapata et al. (2023), Adherence (*n* = 98) [24]	Home exercise program adherence was 15 min per day, 5 days a week (75 min per week) for 1 year. Patients also completed ≥ 8 h of one-on-one supervised PSSE for the first 6 months.	Adherence mentioned in the Methods section of the paper. Patients were given handouts of their HEP. Patients were sent an electronic weekly survey for 52 weeks using Research Electronic Data Capture (REDCap) regarding HEP adherence. Under sub-heading outcomes, HEP adherence for the Exercise group (percentage of prescribed exercises completed from baseline to 6 months and 1-year follow-up).	A weekly two-question survey e-mail queried the total number of days and minutes patients performed their exercises.	Reported as a percentage of the best number of minutes per week. Results demonstrate HEP adherence was 81.6% ± 31.5% from baseline to 6 months and 62.8% ± 37.8% from baseline to 1-year follow up.	Factored into Discussion, highlighting the rate of dropout from the exercise group. Participants preferred electronic data gathering on adherence using REDCap over paperlog. Insights offered by authors about the barriers and challenges with exercise adherence.

## Data Availability

Data sharing is not applicable to this article as no datasets were generated or analyzed.

## Appendix A. PubMed Search Strategy

(((((((((((((((((((treatment adherence and compliance) OR (patient adherence)) OR (client adherence)) OR (patient non-adherence)) OR (patient non adherence)) OR (client compliance)) OR (therapeutic compliance)) OR (patient non-compliance)) OR (patient cooperation)) OR (motivators) AND (physiotherapeutic scoliosis specific exercise)) OR (PSSE)) OR (scoliosis specific exercise)) OR (scientific exercise approach to scoliosis)) OR (SEAS)) OR (Schroth)) AND (adolescent idiopathic scoliosis)) OR (scheuermann)) OR (scheuermann’s)) OR (scheuermanns))
